# Association between cooking fuels and mild cognitive impairment among older adults from six low- and middle-income countries

**DOI:** 10.1038/s41598-022-17216-w

**Published:** 2022-08-18

**Authors:** Lee Smith, Damiano Pizzol, Guillermo F. López Sánchez, Karel Kostev, Hans Oh, Louis Jacob, Nicola Veronese, Benjamin R. Underwood, Laurie Butler, Yvonne Barnett, Mark A. Tully, Ai Koyanagi

**Affiliations:** 1 Centre for Health, Performance, and Wellbeing, Anglia Ruskin University, Cambridge, UK; 2 Italian Agency for Development Cooperation, Gedaref, Khartoum Sudan; 3 Division of Preventive Medicine and Public Health, Department of Public Health Sciences, School of Medicine, University of Murcia, Murcia, Spain; 4 University Hospital of Marburg, Marburg, Germany; 5 Suzanne Dworak Peck School of Social Work, University of Southern California, Los Angeles, CA 90007 USA; 6 Research and Development Unit, Parc Sanitari Sant Joan de Déu, CIBERSAM, ISCIII, 08830 Barcelona, Spain; 7 Faculty of Medicine, University of Versailles Saint-Quentin-en-Yvelines, 78000 Versailles, France; 8 Chair for Biomarkers of Chronic Diseases, Biochemistry Department, College of Science King Saud University, Riyadh, 11451 Saudi Arabia; 9 Department of Internal Medicine, Geriatrics Section, University of Palermo, Palermo, Italy; 10 Cambridgeshire and Peterborough NHS Foundation Trust, The Gnodde Goldman Sachs Translational Neuroscience Unit, University of Cambridge, Cambridge, UK; 11 School of Medicine, Ulster University, Belfast, UK; 12 ICREA, Pg. Lluis Companys 23, 08010 Barcelona, Spain

**Keywords:** Health care, Disease prevention, Public health

## Abstract

There is a small body of evidence suggesting that unclean cooking fuel use may be associated with cognitive decline. However, to date, no study has investigated the association between unclean cooking fuel and mild cognitive impairment (MCI). Thus, we investigated the association between cooking fuel type or ventilation type and MCI among adults aged ≥ 65 years using nationally representative datasets from six low- and middle-income countries. Cross-sectional, community-based data from the World Health Organization (WHO) Study on global Ageing and adult health (SAGE) were analyzed. MCI was defined using the National Institute on Aging-Alzheimer's Association criteria. Unclean cooking fuel referred to kerosene/paraffin, coal/charcoal, wood, agriculture/crop, animal dung, and shrubs/grass. Multivariable logistic regression analysis was conducted to assess associations. Data on 13,623 individuals were analyzed [mean (SD) age 72.8 (11.0) years; 45.5% males]. Unclean cooking fuel (vs. clean cooking fuel) was associated with a significant 1.48 (95% CI = 1.08–2.03) times higher odds for MCI. Having no chimney or hood for cooking ventilation was also associated with significantly higher odds for MCI (OR = 1.88; 95% CI = 1.25–2.84). Unclean cooking fuel use and lack of chimney or hood for cooking ventilation were associated with higher odds for MCI. Findings support the implementation of the United Nations Sustainable Goal 7, which advocates affordable, reliable, sustainable, and modern energy for all, as this may also help reduce MCI and ultimately dementia.

## Introduction

Dementia is a syndrome associated with an ongoing decline of brain functioning^1^, and increases exponentially with age^2^. Globally, approximately 55 million people live with dementia, with over 60% of these people living in low- and middle-income countries (LMICs). As the proportion of older people in the population is increasing in nearly every country, with the greatest increase expected in LMICs, this number is expected to rise to 78 million in 2030, and 139 million in 2050^3^. Dementia is associated with a multitude of negative outcomes. For example, dementia is currently the seventh leading cause of death among all diseases, and one of the major causes of disability and dependency among older people globally^3^. Moreover, dementia has physical, psychological, social and economic impacts, not only for people living with dementia, but also for their caregivers, families and society^3^. However, to date, there is no known cure for dementia; thus, identification of modifiable risk factors for prodromal stages of dementia is considered to be of prime importance to establish interventions to prevent or delay the onset of dementia. Specifically, mild cognitive impairment (MCI) is a preclinical state of dementia with a high conversion rate to dementia (annual conversion rates ranging from 10 to 15% in clinical samples and 3.8% to 6.3% in community-based samples)^4,5^, and is increasingly being recognized as an important “target” for the prevention of dementia. While several potentially modifiable risk factors for MCI have been identified (e.g., physical activity, depression), one potentially important but understudied risk factor especially in the context of LMICs is use of unclean cooking fuel (i.e., kerosene/paraffin, coal/charcoal, wood, agriculture/crop, animal dung, shrubs/grass), which is used by approximately 2.6 million people in LMICs^6^.

Cooking with unclean fuels may increase risk for cognitive decline via systemic inflammation and oxidative stress^7^. Indeed, oxidative damage is involved in the mechanisms of neurodegeneration^8^. Moreover, an increase in air pollutants per se has been found to be associated with smaller total cerebral brain volume and a higher risk of covert brain infarct^9^. Importantly, several studies have found an association between unclean cooking fuel and cognitive impairment. For example, in one longitudinal study including a sample of 8397 middle-aged to older adults from China with 4 years of follow-up, solid cooking fuel use was associated with a greater decline in cognitive score overall, mostly in the episodic memory and visuo-construction dimensions^7^. In another longitudinal study with a 4-year follow-up period also from China including 4145 participants aged ≥ 65 years, biomass fuel use for cooking was associated with a 1.27 (95% CI = 1.02, 1.58) times higher risk for cognitive impairment^10^. Other cross-sectional studies from China^11–13^ and Mexico^14^ have found similar findings. While there is some evidence suggesting that unclean cooking fuel is associated with cognitive impairment, all previous studies only focused on general cognitive decline, which may not necessarily be related to a higher conversion rate to dementia, and none has specifically focused on MCI, which is an established condition known to have a high transition rate to dementia.

Given this background, the aim of the present study was to investigate the association between cooking fuel type including cooking ventilation type and MCI in a large sample of older adults from six LMICs.

## Methods

Data from the Study on Global Ageing and Adult Health (SAGE) were analyzed. Details of the survey methodology have been published elsewhere^15^. In brief, this survey was undertaken in China, Ghana, India, Mexico, Russia, and South Africa between 2007 and 2010. These countries broadly represent different geographical locations and levels of socio-economic and demographic transition. Based on the World Bank classification at the time of the survey, Ghana was the only low-income country, and China and India were lower middle-income countries although China became an upper middle-income country in 2010. The remaining countries were upper middle-income countries. In order to obtain nationally representative samples, a multistage clustered sampling design method was used. The sample consisted of adults aged ≥ 18 years with oversampling of those aged ≥ 50 years. Trained interviewers conducted face-to-face interviews using a standard questionnaire. Those who had a level of cognitive impairment severe enough to preclude the possibility to participate in the survey were not included in the study. Standard translation procedures were undertaken to ensure comparability between countries. The survey response rates were: China 93%; Ghana 81%; India 68%; Mexico 53%; Russia 83%; and South Africa 75%. Sampling weights were constructed to adjust for non-response and the population structure as reported by the United Nations Statistical Division. Ethical approval was obtained from the WHO Ethical Review Committee and local ethics research review boards. Written informed consent was obtained from all participants.

### Mild cognitive impairment (MCI)

MCI was ascertained based on the recommendations of the National Institute on Aging-Alzheimer's Association^16^. We applied the identical algorithms used in previous SAGE publications using the same survey questions to identify MCI^17,18^. Briefly, individuals fulfilling all of the following conditions were considered to have MCI:

- *Concern about a change in cognition*: Individuals who replied ‘bad’ or ‘very bad’’ to the question “How would you best describe your memory at present?” and/or those who answered‘worse’ to the question “Compared to 12 months ago, would you say your memory is now better, the same or worse than it was then?” were considered to have this condition.
- *Objective evidence of impairment in one or more cognitive domains*: was based on a < -1 SD cut-off after adjustment for level of education, age, and country. Cognitive function was assessed through the following performance tests: word list immediate and delayed verbal recall from the Consortium to Establish a Registry for Alzheimer's Disease^19^, which assessed learning and episodic memory; digit span forward and backwards from the Weschler Adult Intelligence Scale^20^, that evaluated attention and working memory; and the animal naming task^21^, which assessed verbal fluency.
- *Preservation of independence in functional abilities*: was assessed by questions on self-reported difficulties with basic activities of daily living (ADL) in the past 30 days^22^. Specific questions were: “How much difficulty did you have in getting dressed?” and “How much difficulty did you have with eating (including cutting up your food)?” The answer options were none, mild, moderate, severe, and extreme (cannot do). Those who answered either none, mild, or moderate to both of these questions were considered to have preservation of independence in functional activities. All other individuals were deleted from the analysis (666 individuals aged ≥ 65 years).
- *No dementia*: Individuals with a level of cognitive impairment severe enough to preclude the possibility to undertake the survey were not included in the current study.

### Cooking fuel

Information on the type of cooking fuel used in the household was obtained by the question “What type of fuel does your household mainly use for cooking?” with the following answer options: gas, electricity, kerosene/paraffin, coal/charcoal, wood, agriculture/crop, animal dung, and shrubs/grass. In line with a previous SAGE publication^23^, this variable was dichotomized as clean fuels (gas, electricity), and non-clean fuels [kerosene/paraffin, solid fuels (coal/charcoal, wood, agriculture/crop, animal dung, shrubs/grass)]. Type of stove and chimney/hood used were further asked only among those who use solid fuels (coal and biomass fuels). Type of stove was asked by the question “In this household, is food cooked on an open fire, an open or closed stove?” This variable was dichotomized as ‘open fire/stove’ or ‘closed stove’^23^. Presence of chimney/hood was assessed with the question “Does the fire/stove have a chimney, hood, or neither?” A dichotomous variable of ‘chimney or hood’ or ‘neither’ was created^23^. Finally, place for cooking was assessed by the question “Where is cooking usually done?” and this variable was dichotomized as ‘In a room used for living or sleeping’ or else (i.e., in a separate room/building used as kitchen, outdoor)^23^.

### Control variables

The selection of the control variables was based on previous literature^12^ and included age, sex, education (≤ primary, secondary, tertiary), wealth quintiles based on income, marital status (currently married/cohabiting, never married, separated/divorced/widowed), setting (rural or urban), smoking (never, current, past), alcohol consumption (never, non-heavy, heavy), physical activity, obesity (body mass index ≥ 30 kg/m^2^ based on measured weight and height), and number of chronic conditions. Consumers of at least four (female participants) or five drinks (male participants) of any alcoholic beverage per day on at least one day in the past week were considered to be ‘heavy’ drinkers. Those who had ever consumed alcohol but were not heavy drinkers were categorized as ‘non-heavy’ drinkers^24^. Levels of physical activity were assessed with the Global Physical Activity Questionnaire and were classified as low, moderate, and high based on conventional cut-offs^25^. Information on 11 chronic physical diseases (angina, arthritis, asthma, chronic back pain, chronic lung disease, diabetes, edentulism, hearing problem, hypertension, stroke, visual impairment) were obtained. The details on the diagnosis of these conditions are provided in Table S1 (Appendix). The number of chronic conditions were summed and categorized as 0, 1, and ≥ 2.

### Statistical analysis

The statistical analysis was done with Stata 14.2 (Stata Corp LP, College station, Texas). The analysis was restricted to those ≥ 65 years as MCI is an age-related condition. The difference in sample characteristics were tested by Chi-squared tests and Student’s *t*-tests for categorical and continuous variables, respectively. Multivariable logistic regression analysis was conducted to assess the association between cooking fuel or cooking ventilation type (exposure) and MCI (outcome). To assess whether there is effect modification by sex in the association between cooking fuel type and MCI, we conducted interaction analysis by including the product term (cooking fuel type X sex) in the regression analysis. The analysis on cooking ventilation type was restricted to those using solid fuels as this information was not obtained for people using other types of cooking fuel. All regression analyses were adjusted for age, sex, education, wealth, marital status, setting, smoking, alcohol consumption, physical activity, obesity, number of chronic conditions, and country. Adjustment for country was done by including dummy variables for each country in the model as in previous SAGE publications^17,26^. The sample weighting and the complex study design were taken into account in all analyses. Results from the regression analyses are presented as odds ratios (ORs) with 95% confidence intervals (CIs). The level of statistical significance was set at *p* < 0.05.

## Results

**Figure 1:**
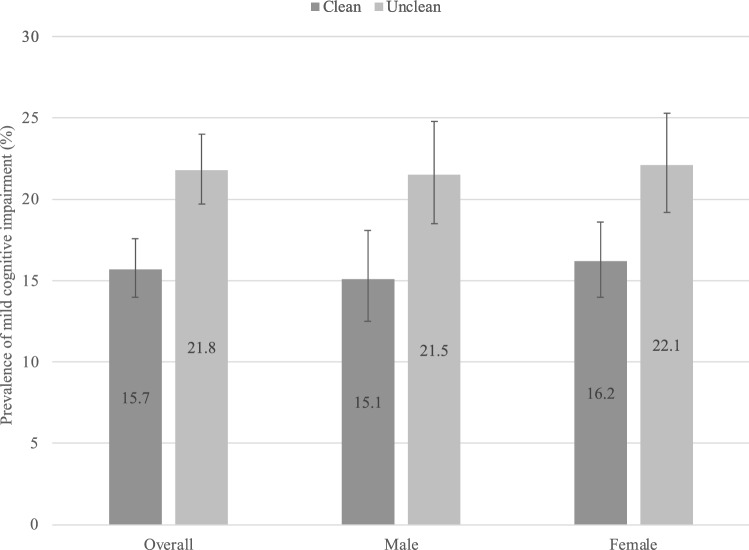
Prevalence of mild cognitive impairment by cooking fuel type (overall and by sex). Bars denote 95% confidence interval.

The final sample consisted of 13,623 individuals aged ≥ 65 years with preservation of functional abilities. The prevalence of MCI was 18.5%, while that of unclean fuel use was 45.0%. Among those who used solid fuels, the prevalence of different cooking ventilation types were: open stove or fire 86.2%; no chimney or hood 53.2%; cooking done in a room used for living or sleeping 11.2%. The sample characteristics are provided in Table 1. The mean (SD) age was 72.8 (11.0) years while 45.5% were males. Factors such as lower levels of wealth, education, and rural setting were strongly associated with unclean cooking fuel use. The sample characteristics by country are provided in Table S2 of the Appendix. The sample sizes per country were: China n = 5094; Ghana n = 1904; India n = 2211; Mexico n = 1179; Russia n = 1820; and South Africa n = 1415. Overall, the prevalence of MCI was higher among those who use unclean cooking fuel compared to those using clean fuel (21.8% vs. 15.7%) (Fig. 1). Similar results were found for males and females. After adjustment for potential confounders, unclean cooking fuel (vs. clean cooking fuel) was associated with a significant 1.48 (95% CI = 1.08, 2.03) times higher odds for MCI (Table 2). There was no significant interaction by sex in this association. In terms of cooking ventilation type, only no chimney or hood was associated with significantly higher odds for MCI (OR = 1.88; 95% CI = 1.25, 2.84) (Table 3).

**Table 1:** Sample characteristics (overall and by cooking fuel).

	Overall (n = 13,623)	Cooking fuel		*P*-value^a^
		Clean (n = 7619)	Unclean (n = 5940)	
**Age (years)**				
Mean (SD)	72.8 (11.0)	72.8 (11.0)	71.6 (10.6)	< 0.001
**Sex**				
Female	54.5	58.0	50.1	< 0.001
Male	45.5	42.0	49.9	
**Education**				
≤ Primary	62.9	41.5	89.0	< 0.001
Secondary	30.4	47.0	10.4	
Tertiary	6.6	11.6	0.6	
**Wealth**				
Poorest	21.3	12.9	31.3	< 0.001
Poorer	21.0	16.4	26.8	
Middle	20.4	21.4	19.2	
Richer	17.5	20.4	13.8	
Richest	19.8	28.8	8.9	
**Marital status**				
Currently married/cohabiting	62.2	60.1	64.9	0.057
Never married	1.2	1.4	1.0	
Separated/divorced/widowed	36.6	38.5	34.2	
**Setting**				
Urban	51.5	79.9	16.8	< 0.001
Rural	48.5	20.1	83.2	
**Smoking**				
Never	62.4	73.4	49.0	< 0.001
Current	29.3	16.7	44.6	
Past	8.3	9.9	6.4	
**Alcohol consumption**				
Never	67.1	59.9	75.7	< 0.001
Non-heavy	30.4	38.0	21.4	
Heavy	2.5	2.2	2.8	
**Physical activity**				
High	36.5	34.0	39.7	0.013
Moderate	25.9	27.6	23.7	
Low	37.6	38.4	36.6	
**Obesity**				
No	89.6	83.6	96.5	< 0.001
Yes	10.4	16.4	3.5	
**No. of chronic conditions**				
0	16.5	12.4	21.5	< 0.001
1	29.6	27.3	32.4	
≥ 2	53.9	60.3	46.1	

Data are % unless otherwise stated.

*SD* standard deviation.

^a^*P*-value was calculated based on Chi-squared tests and Student’s *t*-tests for categorical and continuous variables, respectively.

**Table 2:** Association between type of cooking fuel (or covariates) and mild cognitive impairment estimated by multivariable logistic regression.

Characteristic	OR	95% CI	*P*-value
**Cooking fuel**			
Clean	1.00		
Unclean	1.48	[1.08,2.03]	0.015
Age (years)	1.06	[1.04,1.07]	< 0.001
**Sex**			
Female	1.00		
Male	1.08	[0.79,1.47]	0.642
**Education**			
≤ Primary	1.00		
Secondary	1.05	[0.75,1.46]	0.782
Tertiary	0.44	[0.25,0.77]	0.004
**Wealth**			
Poorest	1.00		
Poorer	0.89	[0.67,1.18]	0.431
Middle	1.27	[0.93,1.73]	0.137
Richer	0.72	[0.54,0.95]	0.019
Richest	0.68	[0.49,0.94]	0.019
**Marital status**			
Currently married/cohabiting	1.00		
Never married	0.80	[0.38,1.65]	0.541
Separated/divorced/widowed	1.12	[0.92,1.36]	0.250
**Setting**			
Urban	1.00		
Rural	1.37	[1.02,1.83]	0.036
**Smoking**			
Never	1.00		
Current	1.20	[0.88,1.63]	0.250
Past	1.25	[0.90,1.74]	0.179
**Alcohol consumption**			
Never	1.00		
Non-heavy	0.91	[0.70,1.18]	0.457
Heavy	1.14	[0.76,1.71]	0.536
**Physical activity**			
High	1.00		
Moderate	0.86	[0.71,1.05]	0.142
Low	1.60	[1.30,1.97]	< 0.001
**Obesity**			
No	1.00		
Yes	1.21	[0.89,1.66]	0.230
**No. of chronic conditions**			
0	1.00		
1	1.32	[0.93,1.86]	0.119
≥ 2	1.47	[1.12,1.92]	0.005

Model is adjusted for all variables in the Table and country.

*OR* odds ratio, *CI* confidence interval.

**Table 3:** Association between cooking ventilation and mild cognitive impairment (outcome) estimated by multivariable logistic regression.

Cooking ventilation	OR	95% CI	*P*-value
**Stove**			
Closed stove	1.00		
Open stove or fire	0.78	[0.60,1.02]	0.074
**Chimney/hood**			
Chimney or hood	1.00		
Without chimney or hood	1.88	[1.25,2.84]	0.003
**Cooking place**			
In a separate room/building used as kitchen or outdoor	1.00		
In a room used for living or sleeping	0.75	[0.43,1.32]	0.317

Models are adjusted for age, sex, education, wealth, marital status, setting, smoking, alcohol consumption, physical activity, obesity, number of chronic conditions, and country.

Sample is restricted to those using solid fuels (coal or biomass fuels).

*OR* odds ratio, *CI* confidence interval.

## Discussion

### Main findings

In this large representative sample of older adults from six LMICs, unclean cooking fuel (vs. clean cooking fuel) was associated with a significant 1.48 (95% CI = 1.08, 2.03) times higher odds for MCI. Associations were similar between males and females. Moreover, when investigating cooking ventilation, the absence of chimney or hood was associated with significantly higher odds for MCI (OR = 1.88; 95% CI = 1.25, 2.84). To the best of our knowledge, this is the first study on unclean cooking fuel use and MCI.

### Interpretation of the findings

The findings of the current study are in line with previous studies on indoor cooking fuel and general cognitive decline from China and Mexico^7,10–14^. Furthermore, in a recent systematic review investigating air pollution and dementia risk including 13 studies, it was observed that greater exposure to particulate matter, nitrogen dioxide, nitrous oxides, and carbon monoxide were all associated with increased risk of dementia^27^.

There are several plausible pathways that may explain the relationship between unclean cooking fuel and MCI observed in our study. These pathways likely act via an increase in indoor air pollutants (i.e., particulate matter) from unclean cooking fuels. Such pollutants induce systemic inflammation and oxidative stress, which may affect the central nervous system through the circulatory system, or translocation by way of the olfactory nerve. The inflammatory reactions in cerebral areas, mediated by cytokines, chemokines, or oxidative stress, may lead to aberrant protein aggregation, impaired neurotransmitter and neurotrophin signaling, neuronal remodeling, and neurodegeneration, which are the leading causes of cognitive impairment^7,28^. Moreover, oxidative damage is involved in the mechanisms of neurodegeneration^8^. Next, an increase in air pollutants has been found to be associated with smaller total cerebral brain volume and a higher risk of covert brain infarct^9^ that are associated with neurological abnormalities, poorer cognitive functioning, and the onset of dementia^7,29^.

Interestingly, previous studies have identified a stronger association between non-clean cooking fuel use and cognitive decline risk in females when compared to males^12,13^, but the present study found no significant interaction by sex in the cooking fuel/MCI association. Previous research has suggested that cognitive functions of females may be more susceptible to hazardous effects of indoor particulate matter due to differences in sex hormones and neuroimmune responses to toxins^12^. However, the present study suggests that this potentially higher female susceptibility to particulate matter may not extend to MCI. Clearly more research is necessary to provide insight on whether gender is an effect modifier in the association between unclean cooking fuel use and MCI.

### Implications of findings

Findings from the present study directly support the implementation of the United Nations Sustainable Development Goal (SDG) 7 “ensure access to affordable, reliable, sustainable and modern energy for all”^30^, which covers reduction of unclean cooking fuel use, and contributes indirectly to reaching 10 out of the 17 SDGs. Specifically, international bodies such as the WHO recommend actions such as government commitment in prioritizing clean-cooking solutions, mobilization of funding to scale up promising enterprises, cross-sectoral collaboration, and monitoring of household energy use^31^. Importantly, unclean cooking fuel use has been found to be associated with multiple negative health outcomes including, for example, respiratory disease, cardiovascular disease, cataract and depression^32–34^. The results of the present study show that eliminating unclean cooking fuel may also have the additional advantage of aiding in the prevention of MCI and possibly dementia.

In addition, the present findings also suggest that the installation of appropriate cooking ventilation devices (i.e., chimney or hood) should be considered among those using solid fuels for cooking as this can remove a greater quantity of pollutants^35^, which in turn may help promote health in the entire household and prevent health problems, including MCI.

### Strengths and limitations

The large representative sample of older adults from multiple LMICs is a clear strength of the present study. However, findings must be interpreted in light of the study limitations. First, the study is cross-sectional in nature and thus, temporal associations cannot be determined. Clearly, more longitudinal studies on this topic are needed. Second, the majority of questions were self-reported. Therefore, it is possible that recall and social desirability bias was introduced into the findings. Next, the present study lacked information on length of exposure to unclean cooking fuel. This information should be considered in future studies as exposure likely builds over the lifetime. For example, autopsy studies from children and young adults living in Mexico City have found associations between exposure to urban air pollution and particulate deposition or inflammation within the brain^28,36^. Next, since the study was not designed to generate clinical diagnoses of dementia, our sample could have included some individuals with mild dementia. However, the prevalence of MCI in our study was within previously reported figures^37^. Furthermore, we were unable to conduct country-wise analyses as stable estimates could not be obtained due to limited sample size and lack of statistical power in each country. Thus, future multi-country studies with larger sample size should assess whether the associations assessed in our study differ by country. Finally, the present study did not collect data on diet. It may be possible that the selection of cooking fuel used is dependent on the food type. Indeed, certain diets have been associated with a higher risk of MCI^38^ and thus may have introduced some bias into the findings.

## Conclusion

In this large representative sample of older adults from multiple LMICs, unclean cooking fuel and a lack of chimney or hood when cooking were associated with a higher risk of MCI. Findings reinforce the importance of the implementation of the United Nations SDG 7, possibly also for the prevention of MCI and ultimately dementia. The installation of chimneys or hoods within home cooking facilities may also have a role in mitigating the deleterious effects of unclean cooking fuel on health.

## Supplementary Information

Supplementary Information 1.

Supplementary Information 2.

## Data Availability

The data that support the findings of this study are available via the WHO website subject to approval. https://apps.who.int/healthinfo/systems/surveydata/index.php/catalog/sage. To request access to licensed datasets, registration in this web is needed.
