# Supplementary material for: Association between cooking fuels and mild cognitive impairment among older adults from six low- and middle-income countries
Source: Sci Rep. 2022 Aug 18;12:14055. doi: 10.1038/s41598-022-17216-w (PMC9388480; doi:10.1038/s41598-022-17216-w)
Supplement: Supplementary file 1 — Supplementary Information 1. [file 41598_2022_17216_MOESM1_ESM.docx]

**APPENDIX**

| **Table S1** Details on the diagnosis of chronic conditions | | |
| --- | --- | --- |
| Condition | (a) Self-reported diagnosis or symptoms | (b) Symptom-based algorithm or other method of diagnosis |
| Angina | Have you ever been diagnosed with angina or angina pectoris (a heart disease)? | Rose questionnaire |
| Arthritis | Have you ever been diagnosed with/told you have arthritis (a disease of the joints, or by other names rheumatism or osteoarthritis)? | NA |
| Asthma | Have you ever been diagnosed with asthma (an allergic respiratory disease)? | NA |
| Chronic back pain | Back pain everyday during the last 30 days. | NA |
| Chronic lung disease | Have you ever been diagnosed with chronic lung disease (emphysema, bronchitis, COPD)? | NA |
| Diabetes | Have you ever been diagnosed with diabetes (high blood sugar)? (not including diabetes associated with a pregnancy) | NA |
| Edentulism | Have you lost all of your natural teeth? | NA |
| Hearing problem | NA | Interviewer observation |
| Hypertension | Have you ever been diagnosed with high blood pressure (hypertension)? | Blood pressure was measured three times with a one-minute interval with the use of a wrist blood pressure monitor (Medistar Wrist Blood Pressure Model S) and the mean value of the three measurements was calculated. Hypertension was defined as having at least one of the following: systolic blood pressure ≥140 mmHg; diastolic blood pressure ≥90 mmHg. |
| Stroke | Have you ever been told by a health professional that you have had a stroke? | NA |
| Visual impairment | Severe/extreme difficulty in seeing and recognizing a person that the participant knows across the road | NA |

For all chronic conditions, we assumed that the individual had the condition if they fulfilled at least one of the following: (a) affirmative answer to self-reported diagnosis or symptoms (b) symptom-based algorithm or other method of diagnosis.
