# Supplementary material for: Association between cooking fuels and mild cognitive impairment among older adults from six low- and middle-income countries
Source: Sci Rep. 2022 Aug 18;12:14055. doi: 10.1038/s41598-022-17216-w (PMC9388480; doi:10.1038/s41598-022-17216-w)
Supplement: Supplementary file 2 — Supplementary Information 2. [file 41598_2022_17216_MOESM2_ESM.docx]

| **Table S2** Sample characteristics by country | | | | | | | |
| --- | --- | --- | --- | --- | --- | --- | --- |
|  |  | China | Ghana | India | Mexico | Russia | South Africa |
| Sample size | N | 5094 | 1904 | 2211 | 1179 | 1820 | 1415 |
| Mean age | Mean (SD) | 72.1 (10.6) | 74.0 (13.8) | 71.2 (9.3) | 73.9 (14.0) | 73.8 (9.8) | 72.7 (14.8) |
| Females | % | 53.2 | 47.5 | 46.8 | 54.4 | 67.6 | 61.0 |
| Survey year |  | 2008-2010 | 2007-2008 | 2007-2008 | 2010 | 2007-2010 | 2007-2008 |
| Mild cognitive impairment | % | 26.4 | 9.7 | 12.5 | 17.3 | 14.8 | 11.3 |
| Unclean cooking fuel | % | 41.4 | 92.8 | 78.4 | 11.1 | 1.6 | 24.0 |

Abbreviation: SD Standard deviation
